# Comparing condylar height/width in patients with and without posterior crossbites

**DOI:** 10.4317/jced.62171

**Published:** 2024-11-01

**Authors:** María José Paniagua, María Rosa Mourelle-Martínez, Marta Muñoz-Corcuera, Begoña Bartolomé-Villar

**Affiliations:** 1 Associate Professor at Faculty of Biomedical Sciences, Department of Clinical Dentistry, Universidad Europea de Madrid, Spain; 2 Professor contracted Doctor of the Department of Dental Clinical Specialties, Complutense University of Madrid, Spain; 3 Tenured Professor at Faculty of Biomedical Sciences, Department of Clinical Dentistry, Universidad Europea de Madrid, Spain; 4 Tenured Professor at Faculty of Biomedical Sciences, Department of Preclinical Dentistry, Universidad Europea de Madrid, Spain

## Abstract

**Background:**

The purpose of this research was to determine the presence of asymmetry of the mandibular condyle in children aged between 7 and 9 years, with a unilateral posterior crossbite, and compare it with a sample of patients without malocclusion.

**Material and Methods:**

The right and left condylar height and width of 401 orthopantomography of children with and without crossbite were measured.

**Results:**

When comparing the height and width of the mandibular condyle in the sample with posterior crossbite we observed that the height and width were almost the same in both condyles. When studying the condylar height in the sample without posterior crossbite, we did not observe differences between both condyles.

**Conclusions:**

The mean condylar height and width in patients with posterior crossbite were higher than that of children without posterior crossbite. This difference was maintained when analyzing the results according to gender and age.

** Key words:**Mandibular condyle, crossbite, orthopantomography, paediatric dentistry, Habets method.

## Introduction

Crossbite is defined as an alteration in the transverse plane where the palatal cusps of the upper posterior teeth occlude lingually from the fossae of the lower posterior teeth in centric occlusion. This occurs due to an imbalance between the width of the maxilla and the mandible, due to maxillary compression or a narrow palate (1).

The etiology can be due to various causes such as environmental, genetic, functional factors, or a combination of these. The most frequent treatments used to avoid this type of malocclusion are varied and include the placement of composite tracks, selective carving, and placement of removable and fixed appliances to expand the upper jaw (2).

Crossbite is considered one of the most prevalent malocclusions in the dental office, especially in children and in growth stages. They can originate from the temporary dentition period, causing an orthopedic imbalance that causes an alteration in the growth and development of the muscles and structures of the face. As a result, a facial asymmetry that can cause alterations in muscles and joints increases the probability of suffering muscle and joint problems in the future (1).

Therefore, it is very important to carry out treatment as soon as possible. About 3 or 4 years of age is the indicated age so that this malocclusion does not worsen (2).

It is considered that the areas that develop the most are the condylar cartilages that can cause the lower jaw to move more in the direction of the affected condyle. For this reason, it can be considered that condylar asymmetry is the reason why asymmetry develops in this type of patient (2,3).

Crossbite has a high frequency of presentation in the dental office and its repercussions at the craniofacial level can produce serious facial asymmetries that increase with age. These posterior crossbites can likely entail changes at the condylar level, mandibular ramus, and coronoid process, which can be quantifiable. For this reason, we have proposed to study the possible morphological changes that posterior crossbites can cause at the condylar level in pediatric patients.

Being able to demonstrate that these changes exist and occur in the early stages of growth would justify the importance of early diagnosis and treatment of these malocclusions to avoid the production of facial asymmetries and possible joint and functional disorders in the future.

The hypothesis for this work was there is an association between posterior unilateral crossbite and condylar asymmetry in childhood patients.

The objective of this research was to determine if there are significant differences in condylar asymmetry in individuals aged 7 to 9 years of both genders, who present unilateral posterior crossbite and compare it with a control group that does not develop this malocclusion. Possible differences in condylar asymmetry between gender and age were also investigated.

## Material and Methods

The study was descriptive, observational, cross-sectional, and retrospective, and was approved by the local Ethics Committee (CIPI 107/17), the investigation has been carried out following the Declaration of Helsinki.

The universe sample was made up of individuals who went to an oral radiodiagnosis clinic to carry out an orthodontic study, including in the records: intra and extraoral photographs, orthopantomography, lateral skull teleradiography, and cephalometric analysis.

The initial sample consisted of a total of 1000 records of patients with orthopantomography and intraoral photographs, made from 2012 to 2015, which included a control group without posterior crossbite and a group of children with a posterior crossbite. For the selection of the sample, inclusion criteria were established as patients of both genders between 7 and 9 years old with posterior crossbite. The exclusion criteria were established as patients with systemic pathologies, without panoramic radiographs or photographs. The inclusion and exclusion criteria for the control group were the same. The investigation was carried out by gender to see if there were statistically significant differences between the sexes, since in the interval of those ages there is growth.

Their parents or guardians had previously signed the informed consent.

After applying the inclusion and exclusion criteria, the final sample consisted of 199 patients with posterior crossbite and 202 patients without posterior crossbite.

With the 16-inch monitor and with the GIMP version 2 program, we captured each digital image of the orthopantomography, using the zoom to increase and then decrease the image by 20% so that the recognition of anatomical structures would be easier to proceed with the measurement. The measure offered by the program is the pixel, which we later transform into millimeters with the help of a ruler shown in the panoramic radiograph.

To carry out the measurements, we used the modified Habets morphological analysis since the Habets method (3), in addition to condylar asymmetry, measures the asymmetry of the ramus and the ramus plus condyle; we only assessed condylar asymmetry, hence we only made the necessary measurements to obtain our results, ignoring the measurement of the ramus and the coronoid process.

We perform the following measurements:

- Point O1: most posterior point of the mandibular condyle. It is the point of greatest convexity of the condylar process.

- Point O2: most posterior point of the mandibular ramus. It is the point of greatest convexity of the mandibular ramus.

- Point D: most anterior point to the head of the mandibular condyle.

- Point E: most posterior point to the head of the mandibular condyle.

- Line A: tangent to points O1 and O2.

- Line B: perpendicular line from A by the most superior point of the condyle 

- Condylar height (CH): it is the length measured from line B to point O1.

- Condylar width (CW): We measured the width of the mandibular condyle by tracing and analyzing the horizontal distance between points D and E.

**Figure 1:**
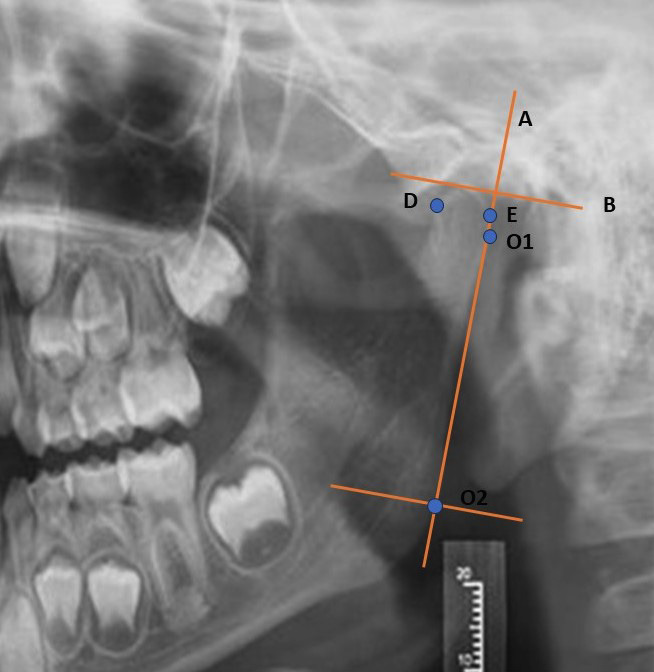
Detail of the condyle with tracings used for the measurement using the modified Habets morphological analysis.

Figure 1 shows a detail of the measurements made.

Statistical analysis: The height and width variables of the condyles were described from the mean, standard deviation, median, interquartile range, and maximum and minimum values for all the patients and the different groups studied (patients grouped by gender and age).

The parametric behavior of the height and width variables of the condyles (Shapiro-Wilk normality tests) was evaluated for all the patients and the different groups studied.

To analyze the possible differences in height and width of the condyles between patients with and without crossbite, Student’s t-tests or Mann-Whitney U tests were used, according to the results obtained from the normality test. The differences in condylar height and width between patients with and without crossbite, and their 95% confidence intervals, were calculated for all patients and the different groups studied (subjects grouped by gender, age, and age and gender).

Statistical significance was considered when the *p-value* was less than 5%. Data analysis was performed with the statistical package STATA IC 14 (Stata Corp., TX, US).

## Results

The sample consisted of 202 children between 7 and 9 years of age of both sexes without posterior crossbite and 199 with it.

-Analysis of the height and width of the condyles in children with unilateral posterior crossbite.

1. Condylar height.

When comparing the height of the mandibular condyle in the sample with posterior crossbite we observed that the height was the same in both condyles (1.05±0.17 mm vs. 1.05±0.16 mm; *p*=0.95).

In the comparison of the height of the condyles by gender, we obtained that it is only in the male sex where there are minimal differences in the condyle height, the contralateral condyle to which the crossbite is slightly higher than the other condyle.

Regarding the age groups, we observed that in 7-year-old boys and girls, the height is identical in both condyles; In the 8-year-old group, the mandibular condyle on the side of the posterior crossbite is slightly higher compared to the contralateral one, while in 9-year-old boys and girls the opposite occurs, being the crossbite condyle slightly smaller relative to the noncrossbite side. None of these differences was statistically significant.

These results are shown in  Table 1.

2. Condylar width.

When comparing the width of the mandibular condyles in patients with a posterior crossbite, we observed that the opposite condyle where the malocclusion occurs is slightly larger compared to the condyle of the crossbite (1.10±0.17 mm vs. 1.09±0.15 mm; *p*=0.92).

When examining by gender, we found that in boys the condyle on the side of the crossbite is larger than on the contralateral side. However, the opposite occurs in the female sex, with the width of the mandibular condyle on the side of the posterior crossbite being smaller than on the contralateral condyle.

In 7-year-old boys and girls, the width of the mandibular condyle on the side of the malocclusion is greater than in the condyle where this pathology does not develop. At 8 years of age, in both sexes, the width of the condyle is also greater on the side where the posterior crossbite develops; in the 9-year-old age group, the results are inverted, obtaining a smaller condylar width in the condyle where the crossbite is located concerning the contralateral one.

None of these differences was statistically significant.

These results are shown in  Table 2.

-Analysis of the condylar height and width in children without posterior crossbite.

1. Condylar height.

When studying the condylar height in the sample without posterior crossbite, we did not observe statistical significance between both condyles. In the female sex, we find that the right condyle is a little higher than the left one.

By age groups, we observed that, in both genders aged 9 years, the mandibular condylar height is greater than in ages 7 and 8 years, and these results are not significant.

2. Condylar width.

In the condylar width in patients without crossbite, it is observed that the left condyle has a lower width compared to the right in the general sample; results are similar to those found in the analysis by gender, obtaining significant differences only for the male sex.

When analyzing the age groups by gender, we found an equal width of both condyles in 8- and 9-year-old males and in 9-year-old females. The right condyle was wider in 7-year-old boys and girls, while the opposite occurred in 8-year-old girls. The differences were statistically significant.

-Comparison of condylar height and width in children with and without unilateral posterior crossbite.

1. Condylar height.

The mean condylar height in children with posterior crossbite was higher than that of children without posterior crossbite (1.05± 0.16 mm vs. 0.96 ± 0.14 mm; *p*<0.0001).

In the analysis by gender, the mean condylar height in the girls with a crossbite was higher than that of the girls without a posterior crossbite making the differences statistically significant.

The results for the total sample are similar when analyzed by sex and by age group, except for the 7-year-old female and male group where the results obtained were practically similar for patients without crossbite and for those who presented it, not observing significant differences for that age group.

These results are shown in  Table 3.

2. Condylar width.

The mean condylar width in boys and girls with posterior crossbite was higher than in patients without posterior crossbite (1.09±0.16 mm vs. 0.97±0.13 mm; *p*<0.001).

When analyzed by age groups the condylar width was significantly greater in all groups of patients who presented posterior crossbite.

When analyzed by gender, we observed how the mean width of the mandibular condyle in girls with malocclusion was higher than in girls without posterior crossbite. These differences were statistically significant.

These results are shown in  Table 4.

## Discussion

Most of the published investigations (4-13) had taken orthopantomography and cephalometry applying the same method in our work as described in 1988 by Habets. Through this method, we can apply this formula to determine the mandibular asymmetries that give patients a unilateral posterior crossbite.

In our sample, we took a control and a study group of patients aged between 7 and 9 years of both genders using orthopantomography to measure condylar height and width on both sides and make a comparison, unlike other authors who did not distinguish between age groups as is the case of Habets (3,7,12,14).

We obtained the largest sample of individuals, consisting of 401 patients, unlike other authors whose sample size was smaller (9,11,12).

We also used as an inclusion criterion the fact that our entire sample lacked systemic pathologies or hereditary syndromes that could predispose the development of facial structures. However, other authors do not indicate the inclusion of this criterion (6).

In short, we can affirm after carrying out this research that untreated crossbite can cause condylar size alterations in childhood patients. The average condylar height and width in boys and girls aged 7-9 years with posterior crossbite were higher than those of boys and girls without posterior crossbite because the contralateral condyle develops more than patients who do not present this malocclusion.

1. Height and width of the condyles in patients with a crossbite.

At the time of checking the condylar height and width in our sample of children with a posterior crossbite, we observed that the height and width were similar in both condyles, thus obtaining slight differences between the mandibular condyle where the posterior crossbite develops and the contralateral.

Similarly, most of the reviewed authors (7,15-23) analyze the condylar height in patients with this type of malocclusion to determine the possible differences that could exist in that parameter, and thus be able to demonstrate the existence of condylar asymmetries in patients with the aforementioned malocclusion.

All of them concluded that, in patients with this malocclusion, an asymmetry was observed in the mandibular condyle on the side where the posterior crossbite developed, causing abnormal growth of the mandibular condyle and ramus. Our results do not confirm the existence of this condylar asymmetry, possibly due to the age of the patients, since the condyle is still growing and developing.

In our research we have made a differentiation by gender, not observing statistically significant differences in the height of the mandibular condyle between both genders; we only obtained minimal differences in condylar height in males, but without significance.

Most of the reviewed authors who include an analysis by gender in their results do not obtain statistically significant differences, with Uysal *et al*. (24) and Kasimoglu *et al*. (25) agreeing with our results.

We have not found in the bibliography any author who analyzed the height of the mandibular condyle in patients with crossbite differentiating by age groups, but rather the majority differentiates between patients with permanent dentition and child patients who are in temporary or mixed first dentition phase (4,6,10).

In this research we differentiate the sample by age, dividing patients between 7 and 9 years of age of both genders. We observed that in 7-year-old boys and girls, the height is the same in both condyles, but in the 8-year-old group, the mandibular condyle on the side of the posterior crossbite is slightly higher than the contralateral one. In 9-year-old boys and girls, the opposite occurs, with the condyle of the crossbite being slightly smaller concerning the non-crossbite side. However, none of these differences was statistically significant.

2. Height and width of the condyles in patients without crossbite.

At the time of checking the condyle height and width in the control group of children without posterior crossbite, we observed that the height was similar in both condyles, thus not obtaining statistically significant differences between the right and left condyles. However, we did find differences in the width between the two condyles, both in the group of patients without crossbite and in the group of children when separating them by gender, and in the group of 7-year-old patients when divided by age (16,19,21).

3. Comparison of the height and width of the condyles in patients with crossbite vs. patients without crossbite.

In our research, when measuring the height and width of the condyle, we obtained significant differences between children who developed the malocclusion and children without it, as did Kilic *et al*. (14), Kiki *et al*. (26), Langberg *et al*. (7) and Vig *et al*. (13), who also measured the parameter in their investigations. However, most authors only measured the height of the condyle without measuring the width of the condyle.

The age groups and gender were separated into two groups. We observed that both boys and girls with posterior crossbite had a higher condylar height than patients without posterior crossbite, these differences being statistically significant. However, in the 7-year-old girls, the height of the condyles was very similar in girls with and without unilateral posterior crossbite.

Authors such as Leonardi *et al*. (21), Halicioglu *et al*. (5), and Celik *et al*. (17) state that there are more asymmetries in the mandibular condyle in adult patients who developed a posterior crossbite than in a control group; therefore, they find that the height of the mandibular condyle is greater in the group of patients who developed this malocclusion. Jing (20), Veli *et al*. (27), and Vig *et al*. (13) obtained similar results, although they did not specify whether it was a unilateral or bilateral crossbite in patients with permanent dentition and first-phase mixed dentition.

4. Limitations

The limitations we encountered in our research were numerous. Obtaining such a large sample of child patients was complex because the informed consent needed to be correctly completed and signed by the parents or legal guardians, and it was not an easy task to find a database of 1000 panoramic radiographs that had sufficient quality to be able to be evaluated. Furthermore, all of them must be taken with the same orthopantomography brand to avoid image distortions.

Another limitation was being able to make a comparison of our sample with that of other authors since in the majority of the studies consulted, the ages of the individuals in the sample were different from those we used in our research.

In conclusion, after analyzing the results from the investigation, we found that both the height and width were significantly higher in boys and girls with posterior crossbites than children without crossbites. This difference was maintained when analyzing the results according to gender and age. The height and width of both condyles in children with unilateral posterior crossbite were similar, with no statistically significant differences being observed either when analyzing the differences between gender and age groups. However, in patients without posterior crossbite, significant differences were found in the width of both condyles, not much in height.

## Figures and Tables

**Table 1:** Condyle height (mm) on the side of the crossbite and the contralateral side in children with crossbite.

CONDYLE HEIGHT (mm) ON THE SIDE OF THE CROSSBITE AND THE CONTRALATERAL SIDE IN CHILDREN WITH CROSSBITE				
	n	Mean	SD	p-value
General				
Condyle contralateral to crossbite	198	1.05	0.17	0.96
Condyle on the side of the crossbite	200	1.05	0.16	
Female				
Condyle contralateral to crossbite	101	1.02	0.17	0.93
Condyle on the side of the crossbite	103	1.02	0.15	
Male				
Condyle contralateral to crossbite	98	1.08	0.17	0.79
Condyle on the side of the crossbite	99	1.07	0.16	
Male and Female 7 years				
Condyle contralateral to crossbite	68	0.96	0.10	0.79
Condyle on the side of the crossbite	68	0.96	0.09	
Male and Female 8 years				
Condyle contralateral to crossbite	68	1.01	0.13	0.96
Condyle on the side of the crossbite	69	1.02	0.13	
Male and Female 9 years				
Condyle contralateral to crossbite	65	1.19	0.18	0.41
Condyle on the side of the crossbite	63	1.18	0.14	

**Table 2:** Condyle width (mm) on the side of the crossbite and the contralateral side in children with crossbite.

CONDYLE WIDTH BY GENDER AND AGE GROUP (mm) ON THE SIDE OF THE CROSSBITE AND THE CONTRALATERAL SIDE IN CHILDREN WITH CROSSBITE				
	n	Mean	SD	p-value
General				
Condyle contralateral to crossbite	198	1.10	0.17	0.92
Condyle on the side of the crossbite	200	1.09	0.15	
Female				
Condyle contralateral to crossbite	101	1.08	0.18	0.70
Condyle on the side of the crossbite	103	1.06	0.16	
Male				
Condyle contralateral to crossbite	99	1.12	0.17	0.71
Condyle on the side of the crossbite	98	1.13	0.14	
Male and Female 7 years				
Condyle contralateral to crossbite	69	1.01	0.14	0.87
Condyle on the side of the crossbite	68	1.02	0.14	
Male and Female 8 years				
Condyle contralateral to crossbite	68	1.03	0.12	0.20
Condyle on the side of the crossbite	69	1.06	0.12	
Male and Female 9 years				
Condyle contralateral to crossbite	63	1.26	0.14	0.07
Condyle on the side of the crossbite	64	1.21	0.13	

**Table 3:** Condyle height (mm) in patients with and without posterior crossbite by gender and age group.

CONDYLE HEIGHT (mm) IN PATIENTS WITH AND WITHOUT CROSSBITE BY GENDER AND AGE GROUP				
	n	Mean	SD	p-value
General				
Children without crossbite	404	0.96	0.14	<0.0001
Children with crossbite	398	1.05	0.16	
Female				
Children without crossbite	202	0.95	0.15	<0.0001
Children with crossbite	199	1.02	0.16	
Male				
Children without crossbite	202	0.98	0.13	<0.0001
Children with crossbite	199	1.08	0.16	
Male and Female 7 years				
Children without crossbite	134	0.95	0.14	0.51
Children with crossbite	136	0.96	0.10	
Male and Female 8 years				
Children without crossbite	134	0.90	0.13	<0.0001
Children with crossbite	136	1.02	0.13	
Male and Female 9 years				
Children without crossbite	133	1.03	0.11	<0.0001
Children with crossbite	126	1.18	0.16	

**Table 4:** Condyle width (mm) in patients with and without posterior crossbite by gender and age group.

CONDYLE WIDTH (mm) BY GENDER AND AGE GROUP IN PATIENTS WITH AND WITHOUT POSTERIOR CROSSBITE				
	n	Mean	SD	p-value
General				
Children without crossbite	404	0.97	0.13	<0.0001
Children with crossbite	398	1.09	0.16	
Female				
Children without crossbite	202	0.94	0.13	<0.0001
Children with crossbite	199	1.07	0.17	
Male				
Children without crossbite	202	0.99	0.12	<0.0001
Children with crossbite	199	1.12	0.15	
Male and Female 7 years				
Children without crossbite	134	0.94	0.12	<0.0001
Children with crossbite	136	1.02	0.14	
Male and Female 8 years				
Children without crossbite	134	0.93	0.13	<0.0001
Children with crossbite	136	1.05	0.12	
Male and Female 9 years				
Children without crossbite	136	1.03	0.11	<0.0001
Children with crossbite	126	1.23	0.14	

## Data Availability

The datasets used and/or analyzed during the current study are available from the corresponding author.
